# Trends in Fruit and Vegetable Consumption Among U.S. Men and Women, 1994–2005

**Published:** 2008-03-15

**Authors:** Heidi Michels Blanck, Cathleen Gillespie, Joel E Kimmons, Jennifer D Seymour, Mary K Serdula

**Affiliations:** Centers for Disease Control and Prevention, National Center for Chronic Disease Prevention and Health Promotion, Division of Nutrition, Physical Activity, and Obesity; Centers for Disease Control and Prevention, National Center for Chronic Disease Prevention and Health Promotion, Division of Nutrition, Physical Activity, and Obesity, Atlanta, Georgia; Centers for Disease Control and Prevention, National Center for Chronic Disease Prevention and Health Promotion, Division of Nutrition, Physical Activity, and Obesity, Atlanta, Georgia; Centers for Disease Control and Prevention, National Center for Chronic Disease Prevention and Health Promotion, Division of Nutrition, Physical Activity, and Obesity, Atlanta, Georgia; Centers for Disease Control and Prevention, National Center for Chronic Disease Prevention and Health Promotion, Division of Nutrition, Physical Activity, and Obesity, Atlanta, Georgia

## Abstract

**Introduction:**

Eating a diet high in fruits and vegetables as part of an overall healthful diet can help lower chronic disease risk and aid in weight management. Increasing the percentage of Americans who consume enough fruits and vegetables every day is part of the *Healthy People 2010* objectives for the nation. Assessing trends in consumption of these foods is important for tracking public health initiatives to meet this goal and for planning future objectives.

**Methods:**

We assessed total and sex-specific changes in daily consumption of fruits and vegetables among 1,227,969 adults in the 50 U.S. states and the District of Columbia who participated in the Behavioral Risk Factor Surveillance System from 1994 through 2005. To estimate changes in consumption according to dietary recommendations that were in place during the years examined, we used geometric mean and the percentage of people eating fruits or vegetables or both five or more times per day. Estimates were standardized for sex, age, and race/ethnicity and analyzed by multivariate regression.

**Results:**

From 1994 through 2005, the geometric mean frequency of consumption of fruits and vegetables declined slightly (standardized change: men and women, −0.22 times/day; men, −0.26 times/day; women, −0.17 times/day). The proportion of men and women eating fruits or vegetables or both five or more times per day was virtually unchanged (men, 20.6% vs 20.3%; women, 28.4% vs 29.6%); however, we found small increases for men aged 18 to 24 years and for women who were aged 25 to 34 years, non-Hispanic black, or nonsmokers. Consumption of fruit juice and nonfried potatoes declined for both sexes.

**Conclusion:**

The frequency of fruit and vegetable consumption changed little from 1994 through 2005. If consumption is to be increased, we must identify and disseminate promising individual and environmental strategies, including policy change.

## Introduction

Fruits and vegetables contain essential vitamins, minerals, fiber, and other bioactive compounds, and a diet high in these foods is associated with lower risk for numerous chronic diseases, including certain cancers and cardiovascular disease (1-3). Because of their low energy density, fruits and vegetables are also beneficial in weight management when eaten as part of a reduced-energy diet (4).

In 1990, the *Dietary Guidelines for Americans* recommended eating at least two servings of fruits and three of vegetables daily (5). In 1991, the National Cancer Institute and the Produce for Better Health Foundation jointly established the national 5 A Day for Better Health Program (6). In 2005, the Centers for Disease Control and Prevention (CDC) became the lead federal agency and health authority for the program, which is a partnership between government, nonprofit organizations, businesses, and communities.

The *Healthy People 2010* objectives for the nation include increasing the percentage of Americans who eat enough fruits and vegetables daily (7). Assessing trends in consumption of these foods is important for tracking public health initiatives to meet this goal and for planning future objectives. An earlier trend analysis that used data from the state-based Behavioral Risk Factor Surveillance System (BRFSS) found that fruit and vegetable consumption by American adults was essentially unchanged from 1994 through 2000 and that a low proportion of Americans ate five or more fruits and vegetables per day (8). However, this analysis did not examine differences in consumption between men and women. Our study updates this earlier work with BRFSS data from the current decade and expands the scope by providing sex-specific results and examining changes in consumption by selected sociodemographic and behavioral characteristics.

## Methods

A continuous telephone survey conducted by state health departments in collaboration with CDC, the BRFSS provides state-specific estimates of behaviors that relate to the leading causes of death among adults in the United States. In each state, random-digit dialing is used to select an independent probability sample of residents aged 18 years or older, and trained interviewers administer identical core questionnaires. Data are weighted by race/ethnicity, age, and sex to reflect the respondents' probability of selection and the state's population. A detailed description of BRFSS methods is available elsewhere (9,10).

We used data from all 50 U.S. states and the District of Columbia that participated in the BRFSS for the years from 1994 through 2005 in which the fruit and vegetable module was part of the core questionnaire: 1994, 1996, 1998, 2000, 2002, 2003, and 2005. We aggregated state estimates to analyze overall and sex-specific consumption over time of fruit juices, whole fruits, green salad, carrots, nonfried potatoes, and "all other" vegetables. We also used BRFSS data to calculate body mass index (BMI), and to evaluate consumption by participation in leisure-time activity and by smoking status.

The BRFSS module on fruits and vegetables has six questions that have remained the same over time. Interviewers begin the module with the following statement: These next questions are about the foods you usually eat or drink. Please tell me how often you eat or drink each one: for example, twice a week, three times a month, and so forth. Respondents are then asked the following questions: 1) How often do you drink fruit juices such as orange, grapefruit, or tomato? 2) Not counting juice, how often do you eat fruit? 3) How often do you eat green salad? 4) How often do you eat potatoes, not including french fries, fried potatoes, or potato chips? 5) How often do you eat carrots? and 6) Not counting carrots, potatoes, or salad, how many servings of vegetables do you usually eat? Participants are not given a definition of serving size. At the end of the fruits and vegetables interview, respondents are asked to report their weight and their height without shoes.

To create an index of fruit and vegetable consumption, we summed the daily frequency of consumption of food items mentioned in the module (11). We calculated total daily fruit consumption from responses to questions 1 and 2 and total daily vegetable consumption from responses to questions 3–6. To calculate consumption in times per day, we divided weekly frequencies by 7, monthly frequencies by 30, and yearly frequencies by 365. For consistency with past analyses, the answer to question 6 was treated as times per day. We calculated BMI as self-reported weight in kilograms divided by height in meters squared and grouped respondents into three categories: normal weight (BMI <25), overweight (BMI 25 to <30), and obese (BMI ≥30) (12).

About leisure-time activity, BRFSS respondents are asked: During the past month, other than during your regular job, did you participate in any physical activities or exercises such as running, calisthenics, golf, gardening, or walking for exercise? We classified smoking status as current smoker, former smoker, and nonsmoker (never smoked). We used the U.S. Census Bureau definition for regions of the United States: Northeast, South, Midwest, and West (13).

A total of 1,394,471 people completed the interviews. We excluded people not reporting race/ethnicity (n = 11,217), education level (n = 2898), smoking status (n = 4138), leisure-time physical activity (n = 5989), and weight or height (n = 58,781) and people who did not answer more than one question in the fruit and vegetable module (n = 103,808) or who reported eating 25 or more fruits and vegetables per day (n = 229). Because some individuals were missing more than one covariate, we had 1,227,969 people in our final sample: 95,571 from 1994; 107,522 from 1996; 130,086 from 1998; 157,179 from 2000; 211,507 from 2002; 224,807 from 2003; and 301,297 from 2005.

To allow comparison with earlier dietary recommendations (14) and previous reports of BRFSS data (7,15), we calculated the percentage of respondents who ate five or more fruits or vegetables or both per day and the mean daily consumption. Because the frequency of consumption was skewed, we calculated geometric mean daily consumption to provide a valid measure of center. Participants who reported not eating any fruits or vegetables were assigned 0.1 times per day to allow for their inclusion in the analysis.

We used state census population estimates for the survey years to weight data for age, race/ethnicity, and sex. Because of the large sample size, we set statistical significance at *P* < .01. To account for the complex sampling design and to report weighted findings, we used SAS 9.1 (SAS Institute, Inc, Cary, North Carolina) and SAS-Callable SUDAAN 9.0 (RTI International, Research Triangle Park, North Carolina). Changes in the geometric mean frequency of daily consumption and in the percentage of respondents eating fruits and vegetables five or more times daily from 1994 through 2005 were standardized to the sex, age, and racial/ethnic distribution of the 2000 BRFSS population. To analyze temporal changes in the geometric mean frequency, we used multivariable regression that controlled for year, age, sex, and race/ethnicity for both linear and quadratic time effects (16). Quadratic trends can be statistically significant but nonlinear over time because of a leveling off or significant change in direction, whereas trends with significant linear and quadratic components demonstrate nonlinear variation along with an overall increase or decrease over time.

## Results

For all survey years combined, 49.2% of respondents were male and 50.8% were female. Seventy-five percent were non-Hispanic white, 9.2% were non-Hispanic black, 10.7% were Hispanic, and 5.1% were of other race/ethnicity. Slightly more than half (56.7%) had at least some college education. By age, 32.7% of respondents were 18 to 34 years; 38.7%, 35 to 54 years; and 28.6%, 55 years or older. Nearly one-quarter of respondents (22.6%) were current smokers, and most (74.3%) had engaged in some leisure-time physical activity in the month preceding their interview. Nearly half (43.6%) reported being of normal weight; 36.4%, being overweight; and 20.0%, being obese.

From 1994 through 2005, the total geometric mean frequency of fruit and vegetable consumption declined slightly, from 3.43 times per day in 1994 to 3.24 times per day in 2005 (standardized change, −0.22 times/day) (Table 1). The geometric mean frequency for men changed from 3.21 to 2.98 times per day (standardized change, −0.26 times/day); women went from 3.66 to 3.50 times per day (standardized change, −0.17 times/day). Overall, total fruit and total vegetable consumption declined slightly. Of the six fruit and vegetable categories, fruit juice (−0.13) and nonfried potatoes (−0.08) showed slight declines in consumption. When stratified by sex, the data showed that men had small declines in the consumption of fruit juice (−0.09), nonfried potatoes (−0.07), and "all other" vegetables (−0.06); women had small declines in the consumption of fruit juice (−0.15) and nonfried potatoes (−0.09). Linear trends were similar to endpoint changes with one exception: the total sample had no significant linear trend for "all other" vegetables. For the total sample, quadratic trends were significant for total fruits; for women, they were significant for green salad. Men had no significant quadratic trends.

The overall prevalence of consumption of fruits or vegetables or both five or more times per day was 24.7%, with no appreciable change between 1994 (24.6%) and 2005 (25.0%) (data not in table). Men's prevalence of this level of consumption did not change significantly from 1994 through 2005 (−0.87 percentage points, *P* = .03) (Table 2). Subgroup analysis, however, found a significant increase (+3.71 percentage points, *P* = .003) among men aged 18 to 24 years and a significant decrease (−3.43 percentage points, *P* = .003) among men aged 55 to 64 years. Significant decreases also were found for non-Hispanic white men (−1.45 percentage points, *P* = .001), men reporting any leisure time physical activity (−1.65 percentage points, *P* = .001), and men residing in the South (−1.81 percentage points, *P* = .01).

Among women, the overall prevalence of eating fruits or vegetables or both five or more times per day did not change significantly from 1994 through 2005 (+0.59 percentage points, *P* = .11). However, several groups had significant increases: women aged 25 to 34 years (+3.65 percentage points, *P* < .001), non-Hispanic black women (+4.08 percentage points, *P* = .0002), and women who were nonsmokers (+1.43 percentage points, *P* = .004) (Table 3). High school graduates showed a significant decrease (−2.18 percentage points, *P* = .001). Significant changes were not observed for women by region or by BMI.

## Discussion

Our findings show that fruit and vegetable consumption among American adults remained relatively stable from 1994 through 2005. The small decrease in vegetable consumption among men was attributable to declines in eating nonfried potatoes and "all other" vegetables and among women, to a decline in eating nonfried potatoes. The decrease among both sexes in total fruit consumption was driven by reduced consumption of fruit juice, not of whole fruit, which remained stable. The decreases in consumption of nonfried potatoes and fruit juice could reflect food transitions among Americans.

Consumption estimates based on abbreviated food frequency questionnaires, such as the BRFSS module, are generally lower than those from studies using other methods (17-19). In fact, BRFSS-based estimates of the proportion of adults eating five or more fruits or vegetables or both per day are consistently lower than estimates based on data from studies that use multiple 24-hour recalls or food records and that include food combinations (e.g., vegetarian pizza, stir fry) and condiments as well as information on serving sizes. The exclusion of fried potatoes in the BRFSS module also contributes to lower estimates. These differences should be considered in interpreting findings.

The new national objectives, which were put into place in 2005, are based on age, sex, and physical activity level and are expressed as cups per day, with the amount that counts as 1 cup being based on form (e.g., 1 small apple; 1 large banana or orange; ½ cup dried fruit; 1 cup chopped, cooked, or canned fruit; 1 cup fruit or vegetable juice; 2 cups raw leafy vegetables; 1 cup raw or cooked other vegetables) (20). Because this analysis reports consumption according to the number of times per day that fruits and vegetables are eaten, the results may over- or underestimate the proportion of people meeting the new objectives. Assuming the accuracy of reporting remains similar across time, however, BRFSS data should correctly reveal trends in consumption frequency, given that the questionnaire has remained the same and that the module has been validated in diverse samples (13).

Considering the health benefits of fruits and vegetables, the lack of an overall increase in consumption is disappointing. Fruits, vegetables, whole grains, and low-fat dairy products are related to high dietary quality and reduced caloric intake when they replace energy-dense, nutrient-poor foods (8,21). Focused interventions can increase fruit and vegetable intake in the short term at the local level (22,23), but the long-term effectiveness of interventions at the state and national levels has not been determined. Reviews of intervention studies to increase fruit and vegetable consumption have found average increases of about 0.6 servings per day (22) with a range of about 0.1 to 1.4 daily servings (23), but few of these studies followed participants for more than 1 year.

National public health goals for fruit and vegetable consumption among all Americans include the *Healthy People 2010* objectives of increasing to 75% the proportion of people aged 2 years or older who eat at least two daily servings of fruit and to 50% the proportion of people aged 2 years or older who eat at least three daily servings of vegetables, with at least one-third being dark green or orange vegetables (7). The most recent *Dietary Guidelines for Americans* recommends that the number of daily servings of fruits and vegetables should reflect one's sex, age, and physical activity level (20). For adults, 3½ to 6½ cups of fruits and vegetables each day are now recommended. Using responses from a single 24-hour recall, Guenther et al (17) recently found that less than 11% of Americans in most sex-age subgroups met the new recommendations in 1999 through 2000, with an exception being older women (17%). Results from a recent trend analysis by Casagrande et al (24) using data from the NHANES surveys, which employ a 24-recall and include serving size and food combinations, were consistent with our finding that the percentage of American adults who eat fruits and vegetables has reduced slightly. They found that, in general, 25% of American adults ate five or more servings of fruits and vegetables daily and that mean daily servings were relatively stable during 1988 through 1994 (3.06) and 1999 through 2000 (3.04) (*P* = .75).

The national findings of Guenther et al (17), Casagrande et al (24), and our analysis underscore the need for broadening interventions beyond increasing individual awareness of the value of fruits and vegetables and education toward changing eating behavior. These broader interventions may require interpersonal, community-level, and environmental approaches. Possibilities include increasing access to fruits and vegetables at daycare centers, schools, universities, and worksites (25,26) and at local farmers' markets through vouchers for seniors (27) and the Special Supplemental Nutrition Program for Women, Infants, and Children (28). Farm-to-school programs (29), school gardening projects (30), and other community projects (31) are also good avenues for encouraging changes in eating behavior.

A new national fruit and vegetable initiative, built on the earlier 5 A Day partnership, is under way. The Fruits and Veggies — More Matters initiative will continue the goal of increasing awareness of the need for people to consume these foods (32,33). With this new initiative comes the need for research and evaluation to identify promising individual and environmental strategies, including policy changes, to increase Americans' consumption of fruits and vegetables.

## Figures and Tables

**Table 1:** Geometric Mean Frequency of Daily Fruit and Vegetable Consumption Among Men and Women, by Year, in the Behavioral Risk Factor Surveillance System (BRFSS), United States, 1994–2005

Food Consumed	Geometric Mean Frequency^a^							Endpoint Change^b^ 1994–2005		

	1994	1996	1998	2000	2002	2003	2005	Change^a^	(95% CI)	*P* Value for Change
**Total**										
**Fruits**										
Juice	0.32	0.32	0.31	0.29	0.25	0.22	0.20	−0.13	(−0.15 to −0.10)	<.001^c^
Whole fruit	0.44	0.45	0.44	0.43	0.43	0.42	0.44	−0.01	(−0.03 to −0.00)	.01
Total	1.06	1.07	1.05	1.03	0.98	0.93	0.96	−0.13	(−0.14 to −0.11)	<.001^c^ ^d^
**Vegetables**										
Green salad	0.28	0.27	0.27	0.28	0.28	0.28	0.28	0.00	(−0.02 to 0.02)	.73
Carrots	0.09	0.10	0.09	0.09	0.10	0.09	0.09	−0.01	(−0.03 to −0.01)	<.001
Potatoes, nonfried	0.22	0.22	0.20	0.18	0.17	0.15	0.14	−0.08	(−0.10 to −0.06)	<.001^c^
All other	0.90	0.88	0.86	0.86	0.86	0.86	0.86	−0.03	(−0.04 to −0.01)	<.001
Total	2.03	2.00	1.96	1.96	1.92	1.91	1.91	−0.11	(−0.12 to −0.10)	<.001^c^
**Total fruits and vegetables**	3.43	3.42	3.36	3.35	3.26	3.21	3.24	−0.22	(−0.22 to −0.21)	<.001^c^
**Men**										
**Fruits**										
Juice	0.34	0.34	0.34	0.32	0.29	0.25	0.25	−0.09	(−0.13 to −0.05)	<.001^c^
Whole fruit	0.38	0.38	0.38	0.36	0.35	0.34	0.37	−0.03	(−0.06 to −0.01)	<.001
Total	0.97	0.98	0.97	0.95	0.90	0.85	0.88	−0.12	(−0.14 to −0.09)	<.001^c^
**Vegetables**										
Green salad	0.26	0.25	0.24	0.24	0.24	0.24	0.24	−0.02	(−0.05 to 0.01)	<.001
Carrots	0.08	0.09	0.08	0.08	0.09	0.08	0.08	0.00	(−0.04 to 0.04)	.004
Potatoes, nonfried	0.23	0.22	0.21	0.19	0.18	0.16	0.15	−0.07	(−0.10 to −0.04)	<.001^c^
All other	0.83	0.80	0.78	0.77	0.76	0.76	0.75	−0.06	(−0.08 to −0.03)	<.001^c^
Total	1.90	1.87	1.81	1.80	1.75	1.73	1.74	−0.17	(−0.18 to −0.15)	<.001^c^
**Total fruits and vegetables**	3.21	3.19	3.12	3.11	3.01	2.94	2.98	−0.26	(−0.27 to −0.24)	<.001^c^
**Women**										
**Fruits**										
Juice	0.31	0.30	0.29	0.26	0.22	0.19	0.16	−0.15	(−0.18 to −0.12)	<.001^c^
Whole fruit	0.52	0.53	0.52	0.50	0.51	0.51	0.54	0.01	(−0.01 to 0.03)	.08
Total	1.15	1.16	1.14	1.11	1.06	1.02	1.03	−0.14	(−0.16 to −0.12)	<.001^c^
**Vegetables**										
Green salad	0.31	0.30	0.30	0.31	0.32	0.34	0.34	0.03	(0.01 to 0.05)	<.001^c^ ^d^
Carrots	0.11	0.11	0.10	0.11	0.11	0.10	0.10	−0.01	(−0.04 to −0.00)	.001
Potatoes, nonfried	0.22	0.22	0.20	0.18	0.16	0.14	0.13	−0.09	(−0.11 to −0.07)	<.001^c^
All other	0.98	0.97	0.95	0.96	0.97	0.97	0.97	0.01	(0.00 to 0.03)	.14
Total	2.16	2.15	2.12	2.12	2.10	2.10	2.11	−0.05	(−0.06 to −0.04)	<.001
**Total fruits and vegetables**	3.66	3.65	3.61	3.60	3.53	3.51	3.50	−0.17	(−0.18 to −0.16)	<.001^c^

CI indicates confidence interval.

a Calculated to provide a valid measure of center because consumption frequency was skewed.

b The change is standardized to the sex, age, and racial/ethnic distribution of the 2000 BRFSS.

c Indicates significant linear trend in model that adjusts for year, sex, age, race/ethnicity, *P* <.01.

d Indicates significant quadratic trend in model that adjusts for year, sex, age, race/ethnicity, *P* <.01.

**Table 2:** Percentage of Men Who Consume Fruits or Vegetables or Both Five or More Times per Day, Behavioral Risk Factor Surveillance System (BRFSS), United States, 1994–2005

Characteristic	Percentage^a^							Endpoint Change 1994–2005		

	1994	1996	1998	2000	2002	2003	2005	Change^a^	(95% CI)	*P* Value
**Total**	20.6	20.8	20.3	20.2	20.2	18.9	20.3	−0.87	(−1.67 to −0.07)	.03
**Age group, y**										
18-24	17.0	17.8	20.9	21.7	20.5	20.3	20.8	3.71	(1.22 to 6.20)	.003
25-34	18.0	19.0	17.7	16.8	18.0	16.9	18.2	−0.37	(−2.11 to 1.37)	.68
35-44	18.5	19.3	17.5	17.6	18.1	16.8	17.2	−1.45	(−3.02 to 0.12)	.07
45-54	21.0	20.1	20.1	18.7	19.0	17.3	19.6	−1.78	(−3.62 to 0.06)	.06
55-64	23.8	21.3	20.9	21.7	20.4	18.9	20.7	−3.43	(−5.66 to −1.20)	.003
≥65	29.5	28.8	27.8	28.7	27.9	25.9	27.8	−1.66	(−3.70 to 0.38)	.11
**Race/ethnicity**										
Non-Hispanic white	20.7	20.7	20.3	20.0	19.5	18.5	19.7	−1.45	(−2.31 to −0.59)	.001
Non-Hispanic black	18.2	18.4	18.0	19.8	21.4	18.9	21.7	3.17	(0.45 to 5.89)	.02
Hispanic	20.3	21.1	21.4	21.0	19.6	18.3	19.8	−0.98	(−4.21 to 2.25)	.55
Other	23.7	25.1	21.7	23.1	26.5	23.9	25.4	1.37	(−2.63 to 5.37)	.50
**Education**										
<High school	18.6	19.4	18.6	18.0	18.1	15.8	18.0	−2.24	(−4.40 to −0.08)	.04
High school graduate	17.4	17.5	17.3	18.0	17.1	16.3	16.8	−1.55	(−2.86 to −0.24)	.02
Some college	20.6	20.7	19.8	19.7	19.4	18.2	19.8	−2.02	(−3.61 to −0.43)	.01
College graduate	25.1	24.8	24.6	23.7	24.4	22.9	24.5	−1.02	(−2.88 to 0.84)	.28
**Smoking status**										
Current smoker	15.6	16.0	15.8	16.2	14.8	14.2	15.6	−0.99	(−2.46 to 0.48)	.19
Former smoker	23.1	23.5	22.3	21.8	22.5	20.7	21.8	−0.66	(−2.40 to 1.08)	.46
Nonsmoker	21.6	21.7	21.6	21.3	21.7	20.4	21.5	−1.36	(−2.59 to −0.13)	.03
**Any leisure time activity**										
Yes	22.6	22.8	22.1	21.9	21.6	20.3	21.8	−1.65	(−2.61 to −0.69)	.001
No	15.0	15.1	15.1	15.0	15.0	13.6	14.8	−0.73	(−2.08 to 0.62)	.29
**Body mass index^b^**										
<25	22.0	22.0	21.6	22.3	22.2	20.9	23.1	0.06	(−1.31 to 1.43)	.93
25 to <30	20.3	20.5	20.0	20.1	19.7	18.5	19.5	−1.28	(−2.48 to −0.08)	.04
≥30	17.9	18.4	18.5	17.3	18.4	17.0	18.2	0.38	(−1.42 to 2.18)	.68
**Region^c^**										
Northeast	20.9	21.8	22.9	21.8	23.0	20.6	21.5	0.41	(−1.35 to 2.17)	.65
Midwest	18.4	17.2	18.0	17.6	16.8	16.6	18.2	−1.24	(−2.87 to 0.39)	.14
South	21.4	20.6	20.4	20.6	20.9	18.9	20.1	−1.81	(−3.16 to −0.46)	.01
West	21.6	23.5	20.3	20.8	19.9	19.7	21.6	0.16	(−1.96 to 2.28)	.88

CI indicates confidence interval.

a The change is standardized to the sex, age, and race/ethnicity distribution of the 2000 BRFSS.

b Weight in kilograms divided by height in meters squared.

c U.S. Census Bureau definition.

**Table 3:** Percentage of Women Who Consume Fruits or Vegetables or Both Five or More Times per Day, Behavioral Risk Factor Surveillance System (BRFSS), United States, 1994–2005

Women	Percentage^a^							Endpoint Change 1994–2005		

	1994	1996	1998	2000	2002	2003	2005	Change^a^	(95% CI)	*P* value
**Total**	28.4	29.1	28.8	29.3	29.5	28.9	29.6	0.59	(−0.14 to 1.32)	.11
**Age group, y**										
18-24	20.6	22.2	22.0	24.1	25.0	24.0	23.5	2.54	(0.13 to 4.95)	.04
25-34	23.2	25.7	24.3	24.2	24.7	25.2	27.2	3.65	(2.04 to 5.26)	<.001
35-44	26.0	26.2	25.6	26.2	27.7	25.9	26.7	0.40	(−1.13 to 1.93)	.60
45-54	29.0	29.0	29.2	28.9	29.0	28.9	29.4	0.47	(−1.33 to 2.27)	.61
55-64	33.6	31.7	31.7	32.5	31.8	31.3	32.1	−1.49	(−3.57 to 0.59)	.16
≥65	39.4	38.5	38.7	39.3	38.1	37.1	37.4	−1.88	(−3.53 to −0.23)	.02
**Race/ethnicity**										
Non-Hispanic white	29.1	29.7	29.9	29.6	29.7	29.4	29.8	0.09	(−0.69 to 0.87)	.82
Non-Hispanic black	22.9	22.3	23.2	24.7	25.8	25.7	27.3	4.08	(1.96 to 6.20)	<.001
Hispanic	28.0	28.8	24.7	29.5	29.3	26.3	28.3	0.42	(−2.83 to 3.67)	.80
Other	30.3	36.4	32.2	35.1	33.1	32.7	32.7	1.55	(−2.78 to 5.88)	.48
**Education**										
<High school	24.0	24.2	22.5	25.2	24.4	24.0	23.9	−1.41	(−3.27 to 0.45)	.14
High school graduate	25.4	25.5	25.4	25.2	25.7	23.9	24.3	−2.18	(−3.41 to −0.95)	.001
Some college	28.6	29.9	29.8	30.2	28.5	29.4	29.8	−0.25	(−1.66 to 1.16)	.73
College graduate	35.5	35.7	35.4	35.2	36.7	35.2	36.3	−0.60	(−2.23 to 1.03)	.47
**Smoking status**										
Current smoker	20.0	20.1	20.6	20.1	19.4	18.6	19.2	−1.37	(−2.84 to 0.10)	.07
Former smoker	32.0	32.7	31.9	31.2	32.5	32.1	31.7	0.61	(−1.04 to 2.26)	.47
Nonsmoker	30.3	31.1	30.6	32.0	32.0	31.2	32.1	1.43	(0.45 to 2.41)	.004
Any leisure time activity										
Yes	31.6	32.5	32.2	32.1	32.4	31.7	32.6	−0.09	(-0.99, 0.81)	.84
No	21.3	21.3	20.8	22.4	21.1	20.5	20.9	−0.73	(-1.91, 0.45)	.22
**Body mass index^b^**										
<25	29.1	29.8	29.4	30.4	30.6	30.6	31.0	0.94	(−0.10 to 1.98)	.08
25 to <30	28.5	29.6	29.4	29.6	30.3	28.9	29.8	1.54	(0.11 to 2.97)	.04
≥30	25.6	25.6	25.9	26.0	25.8	25.0	26.5	0.83	(−0.99 to 2.65)	.38
**Region^c^**										
Northeast	30.3	30.0	31.4	32.4	32.8	32.9	31.8	1.29	(−0.38 to 2.96)	.13
Midwest	27.5	26.4	27.9	26.5	25.9	26.3	27.6	−0.64	(−2.21 to 0.93)	.42
South	26.8	27.1	27.2	27.6	28.6	27.3	27.9	0.64	(−0.58 to 1.86)	.30
West	30.4	34.1	29.8	32.0	31.3	30.3	32.1	1.77	(−0.01 to 3.55)	.05

CI indicates confidence interval.

a The change is standardized to the sex, age, and racial/ethnic distribution of the 2000 BRFSS.

b Weight in kilograms divided by height in meters squared.

c U.S. Census Bureau definition.
